# Do Cupins Have a Function Beyond Being Seed Storage Proteins?

**DOI:** 10.3389/fpls.2015.01215

**Published:** 2016-01-13

**Authors:** Daša Gábrišová, Katarína Klubicová, Maksym Danchenko, Dušan Gömöry, Valentyna V. Berezhna, Ludovit Skultety, Ján A. Miernyk, Namik Rashydov, Martin Hajduch

**Affiliations:** ^1^Department of Developmental and Reproduction Biology, Institute of Plant Genetics and Biotechnology, Slovak Academy of SciencesNitra, Slovakia; ^2^Institute of Virology, Slovak Academy of SciencesBratislava, Slovakia; ^3^Institute of Cell Biology and Genetic Engineering, National Academy of Sciences of UkraineKyiv, Ukraine; ^4^Technical University in ZvolenZvolen, Slovakia; ^5^United States Department of Agriculture, Agricultural Research Service, University of MissouriColumbia, MO, USA

**Keywords:** abiotic stress, flax seeds, ionizing radiation, mass spectrometry, proteomics, Chernobyl

## Abstract

Plants continue to flourish around the site of the Chernobyl Nuclear Power Plant disaster. The ability of plants to transcend the radio-contaminated environment was not anticipated and is not well understood. The aim of this study was to evaluate the proteome of flax (*Linum usitatissimum* L.) during seed filling by plants grown for a third generation near Chernobyl. For this purpose, seeds were harvested at 2, 4, and 6 weeks after flowering and at maturity, from plants grown in either non-radioactive or radio-contaminated experimental fields. Total proteins were extracted and the two-dimensional gel electrophoresis (2-DE) patterns analyzed. This approach established paired abundance profiles for 130 2-DE spots, e.g., profiles for the same spot across seed filling in non-radioactive and radio-contaminated experimental fields. Based on Analysis of Variance (ANOVA) followed by sequential Bonferroni correction, eight of the paired abundance profiles were discordant. Results from tandem mass spectrometry show that four 2-DE spots are discordant because they contain fragments of the cupin superfamily-proteins. Most of the fragments were derived from the N-terminal half of native cupins. Revisiting previously published data, it was found that cupin-fragments were also involved with discordance in paired abundance profiles of second generation flax seeds. Based on these observations we present an updated working model for the growth and reproductive success of flax in a radio-contaminated Chernobyl environment. This model suggests that the increased abundance of cupin fragments or isoforms and monomers contributes to the successful growth and reproduction of flax in a radio-contaminated environment.

## Introduction

Plant growth in radio-contaminated environments is characterized by an increased frequency of DNA mutations (Møller and Mousseau, 2015). Changes in DNA are not, however, always manifest at the protein level (Hajduch et al., 2010), and therefore proteomics studies are desirable to further investigate plant growth and reproduction. In this context, our group has systematically compared seed proteomes from plants grown in both radio-contaminated and non-radioactive areas near Chernobyl since 2007 (Rashydov and Hajduch, 2015). In the first plant generations we saw limited effects of growth in a radio-contaminated environment on mature soybean (Danchenko et al., 2009) and flax (Klubicová et al., 2010) seed proteomes, and detected changes in the abundance of enzymes involved in glycine betaine synthesis. Glycine betaine is involved in protection of human blood against IR (Monobe et al., 2005). Recently we speculated that glycine betaine might be also be involved in plant responses toward a radio-contaminated environment (Rashydov and Hajduch, 2015).

To complement these data, seed development was studied in a second generation of plants grown in the Chernobyl area. Seed filling is the developmental phase where embryos and endosperm undergo morphological (Norton and Harris, 1975) and metabolic (Ruuska et al., 2002) changes, and during which most storage reserves accumulate. It is thus important to investigate seed filling as a prelude to modifying levels of economically important storage polymers such as protein and oil. Flax seeds, for instance, contain 45% of their mass as oil, of which 70% comprises polyunsaturated fatty acids. Our previous studies revealed that growth in the radio-contaminated Chernobyl environment altered oil content in mature flax (Klubicová et al., 2013) and soybean (Klubicová et al., 2012) seeds. The proteomic study of seed filling in Chernobyl showed altered abundance in proteins associated with pyruvate biosynthesis, ethanol oxidation, and of the multifunctional enzyme isocitrate dehydrogenase in flax (Klubicová et al., 2013), decreases in levels of the β-conclycinin SSP plus an altered abundance of proteins associated with carbon metabolism, fatty acid biosynthesis, and the tricarboxylic acid cycle in soybeans (Klubicová et al., 2012).

Overall, we saw limited effects of growth in a radio-contaminated environment on mature and developing soybean and flax seeds (Rashydov and Hajduch, 2015). Limited effects of IR on animal cell growth and development were also noted during studies of animal cells (Park et al., 2006; Guipaud et al., 2007). Plant cells are generally considered to be more resistant to IR than animal cells, due primarily to differences in structure and metabolism (Arena et al., 2014).

The alterations in seed proteomes described in the first two flax generation (Klubicová et al., 2010, 2013) might have been the result of environmental factors other than IR (pests, soil, weather etc). To minimize this possibility, herein we analyzed seed filling by a third generation of flax plants in order to detect alterations common to at least two plant generations.

## Materials and methods

### Experimental fields

The radio-contaminated experimental field, located approximately 5 km from the Chernobyl Nuclear Power Plant (CNPP) near the village Chistogalovka, had a soil radioactivity of 20,650 ± 1050 Bq.kg^−1^ of ^137^Cs, and 5180 ± 550 Bq.kg^−1^ of ^90^Sr. The control experimental field is directly in Chernobyl town, and had a soil radioactivity of 1414 ± 71 Bq.kg^−1^ of ^137^Cs and 550 ± 55 Bq.kg^−1^ of ^90^Sr (Rashydov and Hajduch, 2015). Overall the Chernobyl area is characterized by sod-podzol soil comprising 12% clay and 2% organic material, with a pH value of 5.5.

### Plant material and protein isolation

**Figure 1:**
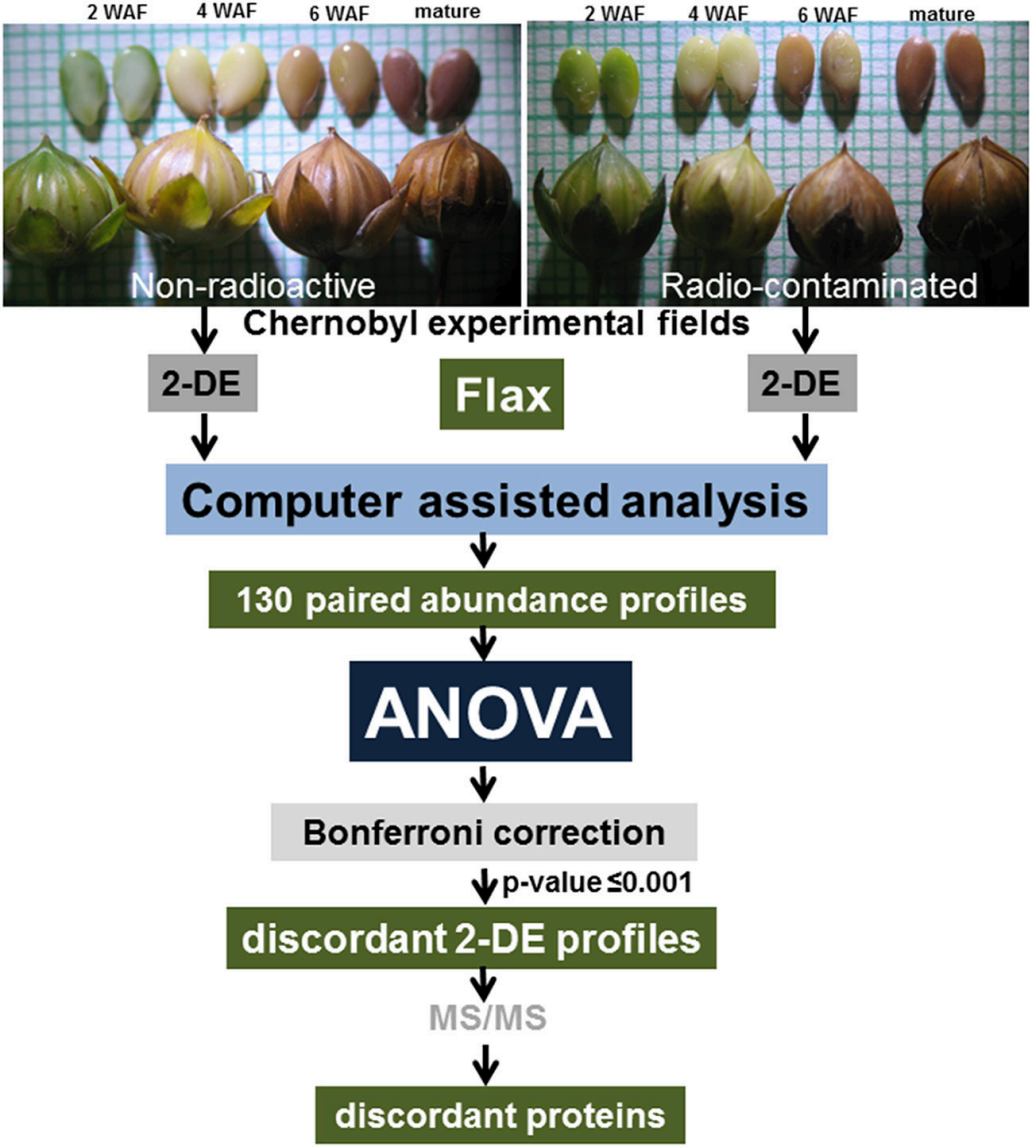
**Experimental design**. Flax seeds were harvested at 2, 4, and 6 weeks after flowering, and at maturity, from plants grown in experimental fields established in the Chernobyl area. Isolated protein samples were resolved by 2-DE and spots were visualized by staining whole gels with Colloidal Coomassie Blue. Images of stained gels were digitized, and computer-assisted image analysis was used to quantify changes in abundance between paired spots. A total of 130 paired spot abundance profiles were established and ANOVA analysis followed by Bonferroni correction was used to idenetify protein spots discordant in abundance patterns.

Flax (*Linum usitatissimum, L*.) seeds of the local variety Kyivskyi were sown in the experimental fields in the spring of 2007. In the current study, flax seeds were harvested at 2, 4, and 6 weeks after flowering (WAF), and at seed maturity (Figure 1) during the 2009 growing season (the third generation of growth in the Chernobyl area). It should be noted that in 2007, when these experiments were initiated and the first flax generation was harvested, the non-radioactive experimental field was located near the village Zhukin, approximately 100 km from the CNPP. This field had soil radioactivity levels of 1350±75 Bq.kg^−1^ for ^137^Cs and 490±60 Bq.kg^−1^ for ^90^Sr (Rashydov and Hajduch, 2015).

Proteins were isolated in biological triplicate as previously described (Klubicová et al., 2010), with minor modifications. Harvested seeds were pooled and divided into three groups, each representing a biological replicate. For each replicate, 200 mg of seeds were ground using a mortar and pestle in liquid nitrogen. Seed powder was mixed immediately with extraction medium [50% (v/v) phenol, 50 mM Tris-HCl, pH 8.8, 5 mM EDTA, 0.45 M sucrose, and 0.2 % (v/v) 2-mercaptoethanol]. After thawing, proteins were extracted using a rotating platform for 30 min at 4°C. The protein-containing phenol phase was separated by centrifugation at 5000 × g for 10 min at 4°C. Proteins were precipitated by addition of 5 volumes of ice-cold 0.1 M ammonium acetate in methanol at −20°C for 16 h. Precipitated proteins were collected by centrifugation at 5000 × g for 10 min. Protein pellets were washed twice with 0.1 M ammonium acetate in methanol, and once each with 80% acetone, and 70% ethanol. Proteins were stored as precipitates at −80°C prior to analysis.

### Two-dimensional gel electrophoresis and image analysis

Before two-dimensional gel electrophoresis (2-DE), proteins were dissolved in isoelectric focusing (IEF) buffer [8 M urea, 2% (w/v) CHAPS, 2 M thiourea, 50 mM DTT, and 2% (v/v) Triton X-100]. Protein concentrations were determined according to Bradford (1976). Protein solutions (500 μg) were clarified by centrifugation before IEF. Carrier ampholytes (pH 3–10; 2% v/v) were added and the total volume was adjusted to 315 μL with IEF buffer. Samples were added to immobilized pH gradient (IPG) strips (pH 5 to 8, 17 cm, Bio-Rad, Hercules, CA). The IEF was performed using a Protean IEF Cell (Bio-Rad, Hercules, CA) programmed for active rehydration for 10 h at 50 V followed by a three-step focusing protocol: (a) 100 Vh at 100 V, (b) 500 Vh at 500 V, and (c) 70,000 Vh at 8000 V. After IEF, the IPG strips were incubated in 4 mL of SDS equilibration solution (0.5 M Tris, pH 8.8, 30% glycerol, 6 M urea, and 2% SDS) plus 2% (w/v) DTT for 15 min. This was followed by incubation for an additional 15 min in SDS equilibration solution containing 2.5% (w/v) iodoacetamide. The IPG strips were then rinsed in running buffer [25 mM Tris, 0.1% (w/v) SDS, and 192 mM glycine], placed onto a 12% SDS gel, and overlaid with 0.5% (v/w) agarose in running buffer (with addition of 0.002% bromphenol blue as the tracking dye). Electrophoresis was performed using a Protean II xi Cell (Bio-Rad, Hercules, CA), at 10 mA current over night or until the tracking dye migrated out of the gel.

After 2-DE, protein gels were rinsed three times for 15 min in deionized water to remove SDS. The gels were then stained overnight in Colloidal Coomassie Blue (CCB) solution [20% (v/v) ethanol, 8% (w/v) ammonium sulfate, 1.6% (v/v) phosphoric acid, and 0.08% (w/v) Coomassie Brilliant Blue G-250], using a rotary agitator (Labnet, Rutland, UK). After CCB staining, gels were rinsed with water, and digitally-imaged using a GS-800 Calibrated Densitometer (Bio-Rad, Hercules, CA) at 16 bit grayscale and 300 dpi settings. Quantitative image analysis was accomplished using ImageMaster 2D Platinum 4.9 software (GE Healthcare, Uppsala, Sweden). The abundance of 2-DE protein spots, expressed as spot volume (V), is a combination of spot intensity and spot area. The volume of each protein spot analyzed was normalized (%V) to compensate for slight differences in sample loading and gel staining.

### Establishing protein spot profiles through flax seed filling

To establish abundance profiles, 2-DE gels of each developmental stage (Supplementary Image 1) were individually matched to the reference 2-DE gel in biological triplicate as described previously (Klubicová et al., 2013). Briefly, the reference gel was established by the analysis of pooled samples from all investigated stages (Klubicová et al., 2013). Protein spot profiles were established by grouping matched protein spots into subclasses. To be included in the analysis, each 2-DE spot had to be presented in at least two biological replicates of three developmental stages in both datasets, i.e., from non-radioactive and radio-contaminated experimental fields. Using this approach any 2-DE spots present only in one dataset were excluded from this study, and common abundance profiles for 130 2-DE spots were established across all developmental stages in both experimental fields (Supplementary Tables 1A–D). To identify discordance in protein abundance patterns during seed development from plants grown in non-radioactive and radio-contaminated Chernobyl areas, data were tested separately using Two-way analysis of variance (ANOVA). The effects of radio-contaminated environment and seed developmental stage were considered fixed. Because of multiple testing, probabilities resulting from the *F*-tests of ANOVA were subjected to sequential Bonferroni correction (Quinn and Keough, 2002). Using this approach, we conclude that eight proteins were differentially abundant during seed filling by plants grown in radio-contaminated and non-radioactive experimental fields, when a cut-off-value of *P* < 0.001 was used (Supplementary Tables 1E,F).

### Protein identification by tandem mass spectrometry

Proteins in eight spots were shown to be differentially abundant when excised from 2-DE gels and washed with 50 mM ammonium bicarbonate in 50% acetonitrile to remove the CCB stain, then dehydrated in 100% acetonitrile. The gel plugs were rehydrated with digestion solution (20 μg of modified sequencing grade trypsin from Promega in 1 mL of 50 mM ammonium bicarbonate) overnight at 37°C. Tryptic peptides were extracted, lyophilized, and stored at −80°C prior to analysis. Tryptic peptides were subjected to data-independent MS^E^ analysis. The MS^E^ method uses alternate scans at low and high collision energies and is able to obtain full-scan mass data for both precursors and fragments in a single run (Plumb et al., 2006). Peptides were directly injected onto a Symmetry C18 trap column (20 mm length, 180 μm diameter, 5 μm particle size). After 3 min of desalting/concentration by 2% acetonitrile containing 0.1% formic acid at a flow rate 10 μL·min^−1^, peptides were eluted into an analytical nanoAcquity UPLC column (BEH 130 C18, 100 μm x 150 mm, 1.7 μm particle size) at a flow rate of 350 nL/min. A 20 min gradient of 7–40% acetonitrile with 0.1% formic acid was used, at a flow rate 350 nL·min^−1^ (10–45% B in 40 min; A = water with 0.1% formic acid, B = acetonitrile containing 0.1% formic acid). The analytical column was mounted directly to the PicoTip emitters (360 μm outer diameter, 20 μm inner diameter, 10 μm tip diameter; New Objective, USA) and samples were nanosprayed (3.4 kV at 70°C) to the quadrupole time-of-flight mass spectrometer (Q-TOF Premier, Waters, UK). The external mass calibrant Glu-1-fibrinopeptide B (400 fmol/mL) was sampled every 30 s and was infused though the Lock-mass source at a flow rate of 350 nL/min. To collect calibrant data, a collision energy of 20 eV was used. To collect MS^E^ data, alternating low (4 eV) and elevated collision energy modes were used. The collision energy was ramped from 20 to 35 eV during each integration in the elevated energy mode. Ions with 50–1950 m·z^−1^ were detected in both channels. Quadrupole mass profile settings allowed efficient deflection of masses < 400 m·z^−1^ in low energy mode, enabling filtering of contaminating ions. The spectral acquisition scan rate was 0.8 s, with a 0.05 s inter-scan delay.

### Data processing

The instrument used for MS analysis was equipped with MassLynx software 4.1 (Waters, USA). The MS^E^ data were processed using the Protein Lynx Global Server v. 3.0 (PLGS, Waters, UK) with the original search algorithm. Peak extraction included smoothing, centroiding, and deisotoping. Noise filtering thresholds were set at 1200 counts (low energy 150 counts, high energy 20 counts). The correlations of precursors and fragment ions were achieved by time alignment of chromatographic elution profiles. Searches were performed against 43,484 *Linum* sequences downloaded in October 2014 from Phytozome (Wang et al., 2012). Common contaminants such as keratin (UniProt P04264) and trypsin (Uniprot P00761) were added to this database. The full-length RuBisCO LSU sequence (Uniprot C7A612) was also appended. Database search parameters were; (i) a minimum of three consecutive product ion matches per peptide, (ii) a minimum of seven total product ion matches per protein, and (iii) a mass accuracy of 20 ppm for precursor ions and 40 ppm for product ions. The search was performed with carbamidomethylation of Cys as a fixed modification, deamidation of Asn/Gln, and oxidation of Met as variable modifications. One missed tryptic cleavage site was accepted and maximum 4% false positive rate against 1x randomized database and was applied at the individual peptide level.

To accept protein identification, additional thresholds were established; (i) a minimum of two peptides must match the protein sequence, and (ii) a PLGS score of >50 was necessary. If MS provided multiple protein identifications for a single 2-DE spot (Supplementary Table 2), the one with the greatest number of peptides was selected. In a case of the same number of identified peptides, the ID with the highest PLGS score was accepted. The PLGS score is a statistical measure of accuracy of assignment, and was calculated during the database search using a Monte Carlo algorithm.

## Results

To minimize spot overlap (Campostrini et al., 2005) 17 cm narrow range IPG strips were used (Supplementary Image 1). Identified differentially abundant 2-DE spots did not provided multiple protein identifications, except for expected contaminants such as keratin or trypsin (Supplementary Table 2). The application of our proteomics approach for comparative analysis of the third generation of flax seed-filling in non-radioactive versus radio-contaminated Chernobyl areas allowed us to establish paired abundance profiles for 130 2-DE spots (Figure 1). The concordance or discordance of these profiles was analyzed using ANOVA (Figure 1).

### Discordant abundance patterns during flax seed filling

**Figure 2:**
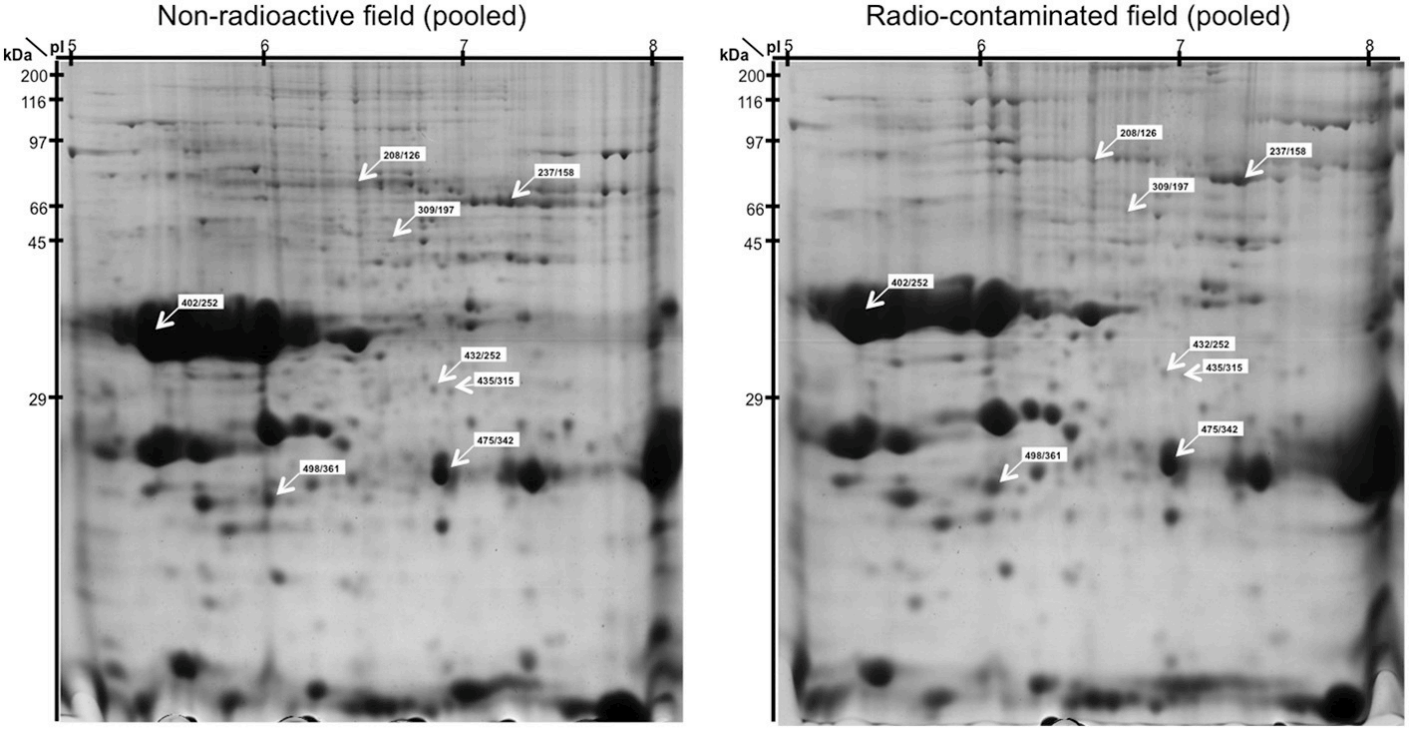
**Reference (pooled) 2-DE gel images of protein spots from developing and mature flax seeds harvested from non-radioactive and radio-contaminated experimental fields in the Chernobyl area**. The positions of the eight 2-DE spots (spot number from non-radioactive/radio-contaminated fields) with discordant abundance during seed development are indicated.

Using ANOVA, a total of eight paired 2-DE spots were significantly discordant when comparing results from plants grown in the non-radioactive versus radio-contaminated plots (Supplementary Table 1). These spots were assigned on reference 2-DE gels from non-radioactive and radio-contaminated Chernobyl areas (Figure 2). All of these discordant spots were identified using MS/MS (Table 1) and sorted to functional categories according to Bevan (Bevan et al., 1998). The most abundant functional group contained four proteins associated with destination and storage, followed by two proteins annotated as having an involvement with energy and two proteins with disease/defense (Table 1).

**Table 1:** **Differentially abundant proteins**.

**Protein description**	**Accession number**	**Localization**	**Spot ID control**	**Spot ID radioactive**	**PLGS Score**	**Cov/Pep**	**Theor Mass/pI**	**Exp Mass/pI**
**ENERGY**								
**Fermentation**								
Alcohol dehydrogenase 1	Lus10010506	Cytoplasm	309	197	412	32,6/12	41/6,1	45/6,5
**Photosynthesis**								
Ribulose-1,5-bisphosphate carboxylas	C7A612	Cytoplasm	208	126	1567	17/7	47/6,1	58/6,4
**PROTEIN DESTINATION AND STORAGE**								
**Storage proteins**								
RmlC-like cupins superfamily protein	Lus10022927	ER	402	252	5679	35.6/32	54/6,0	29/5,5
RmlC-like cupins superfamily protein	Lus10003554	ER	475	342	918	25.1/17	55/5,5	19/6,7
RmlC-like cupins superfamily protein	Lus10003554	ER	498	195	464	8.3/5	56/5,6	17/6,1
cupin family protein	Lus10022070	ER	237	158	1732	19.6/17	60/7,5	53/7,0
**DISEASE/DEFENSE**								
**Detoxification**								
Glutathione S-transferase TAU 19	Lus10042468	Cytoplasm	432	311	1012	21,3/6	25/6,4	23/6,6
Dehydroascorbate reductase 2	Lus10040320	Cytoplasm	435	315	3269	32,4/15	55/5,9	23/6,7

*The Table includes; protein description, accession number, spot IDs from both datasets, Protein Lynx Global Server Score, percent of sequence coverage/number of matched peptides, and deduced/experimental MW/pI values*.

Among the eight proteins identified from 2-DE spots with discordant paired abundance profiles, four were cupins (Table 1). The experimental Mr and pI values of all four proteins identified as cupin superfamily members differ from the cDNA-deduced MW and pI values, suggesting that they are fragmented cupins (Table 1). In two instances, peptides mapping to the N-terminal half of the cupin sequences were detected (Supplementary Table 3A). In 2-DE spots (402/252) multiple peptides were localized throughout whole cupin sequences (Supplementary Table 3B). Peptides comprising complete full-length cupin sequences were found in only 2-DE spot 237/158 (Supplementary Table 3C).

### Protein accumulation during seed filling in the radio-contaminated area

**Figure 3:**
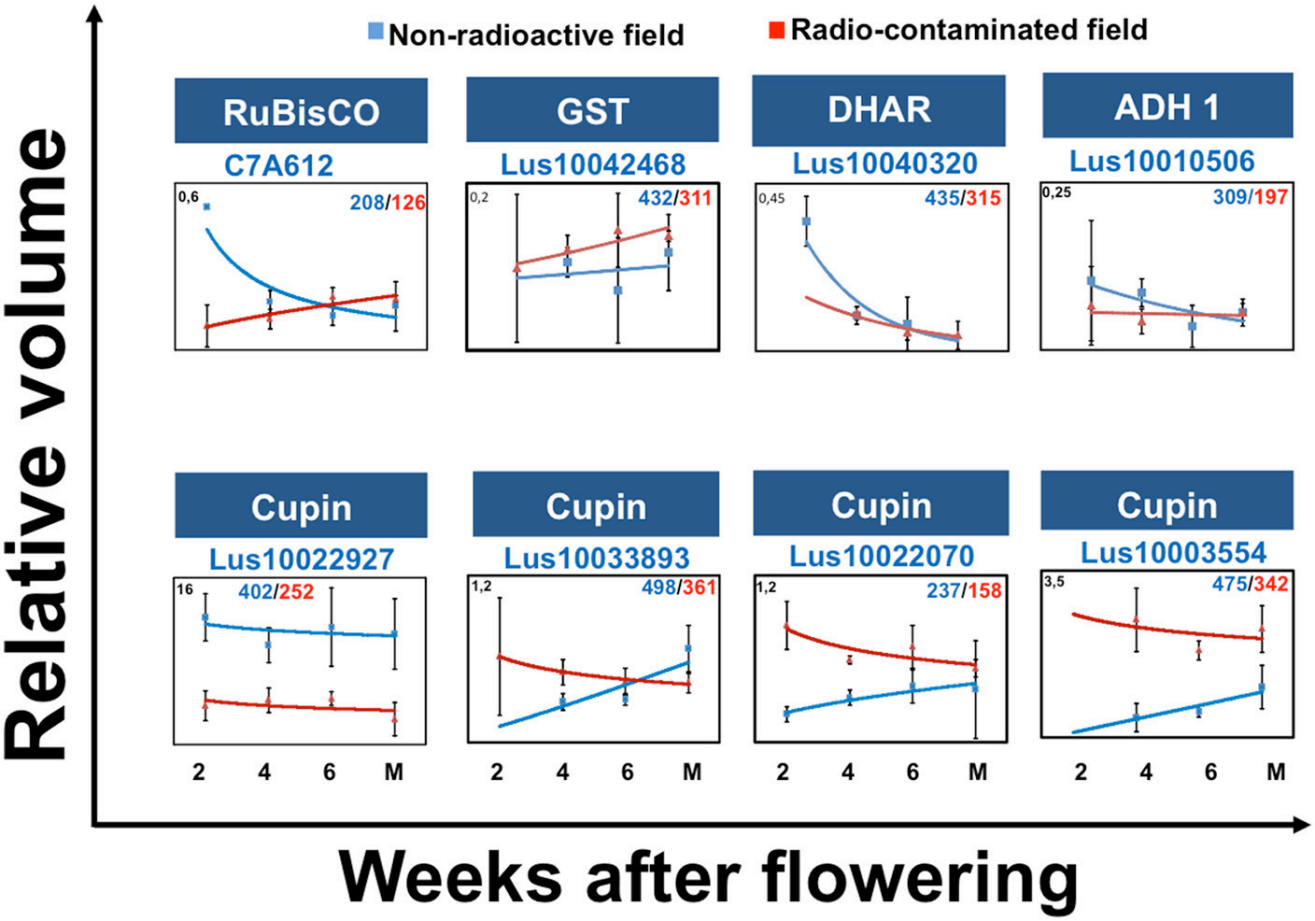
**Discordant protein abundance during seed development**. The graphs show: protein abundances during seed filling at 2, 4, 6 weeks after flowering (WAF) and mature stage in non-radioactive (blue) and radio-contaminated (red) Chernobyl experimental fields. Graph also show the standard deviations, relative volume values (Y axis), protein name, accession number, spot number in the non-radioactive experimental field (blue)/spot number in the radio-contaminated experimental field (red).

To gain insight into alterations in the levels of eight proteins with discordant developmental profiles during seed filling, joint abundance profiles were plotted as nonlinear graphs (Figure 3). Analysis of these graphs revealed that the abundance 2-DE spot 237/158, in which full-length cupin was detected, increased during flax seed filling in the radio-contaminated Chernobyl area (Figure 3). Similarly, 2-DE spot 475/432 containing fragmented cupins increased during flax seed filling in the radio-contaminated Chernobyl area (Figure 3).

The abundance of 2-DE spot 208/126, containing ribulose-1,5-bisphosphate carboxylase oxygenase (RuBisCO) large subunit (LSU), decreased during seed filling in radio-contaminated area (Figure 3). The abundance of dehydroascorbate reductase two detected in 2-DE spot 435/315 increased in abundance during early stages of seed filling (Figure 3).

## Discussion

The aim of this study was to use 2-DE joint abundance profiles to characterize protein accumulation during seed filling of flax grown for a third generation in either non-radioactive or radio-contaminated environments. It is notable, that only 6% of the established paired-abundance profiles were found differentially abundant when comparing seed filling in non-radioactive and radio-contaminated fields in the Chernobyl area. This confirms our previous studies on mature seeds, where only 9% of soybean (Danchenko et al., 2009) and 5% of flax (Klubicová et al., 2010) proteins were found differentially abundant when comparing seeds harvested from non-radioactive and radio-contaminated fields in the Chernobyl area (Rashydov and Hajduch, 2015).

Protein abundance profiles were used previously to characterize seed filling in soybean (Hajduch et al., 2005), canola (Hajduch et al., 2006), and castor (Houston et al., 2009). Additionally, abundance profiles established for proteins during seed filling of the reference dicot plant *Arabidopsis thaliana* were used to asses concordance in expression of transcript and proteins (Hajduch et al., 2010). The study established 319 transcript/protein joint abundance profiles and showed concurrence for 56% of these pairs (Hajduch et al., 2010).

### Cupins—more than seed storage proteins?

The cupins comprise a functionally diverse protein superfamily occurring in both prokaryotic and eukaryotic organisms (Khuri et al., 2001) and sharing a conserved β-barrel fold that was originally observed in the germin seed proteins (Dunwell et al., 2004). Discovery of the β-barrel motif in the slime mold spherulin protein was seminal in establishing the cupin superfamily (Dunwell, 1998), which furthermore includes a variety of members including the sucrose binding proteins (Pirovani et al., 2002), auxin binding proteins (Woo et al., 2002), seed storage globulins (Dunwell, 2005), and the dioxygenase enzymes (Dunwell et al., 2001). Cupins are also major seed allergens (Mills et al., 2002; Breiteneder and Radauer, 2004). The expression of cupins occurs during floral induction (Staiger et al., 1999) and embryogenesis (Neutelings et al., 1998), and after exposure to a variety of both non-biological (Hurkman and Tanaka, 1996; Hamel et al., 1998; Vallelian-Bindschedler et al., 1998) and biological stress conditions (Thordalchristensen et al., 1997).

While specific functional roles remain enigmatic, the synthesis and accumulation of cupins and cupin-related proteins is a normal component of seed development (Dunwell et al., 2004; Koshino et al., 2008; Laudencia-Chingcuanco and Vensel, 2008). Almost half of the peptides identified in this study were fragments of cupins. There is precedent for proteolytic fragmentation of cupin-family proteins in seeds (e.g., Xiang et al., 2004; Wan et al., 2007), and Castillo et al. (2005) present an intriguing model where a proteolytic fragment from a cupin-family protein is involved in control of gene expression during pea seed development. However, it was shown that germin-like proteins (GLP), which belong to cupin-superfamily, might be expressed as several isoforms and monomers. For instance, proteome analysis of lemon fruits indicated that GLP might be expressed as several isoforms and monomers ranging from 120 to 20 kDa (Pignataro et al., 2010). Furthermore, electrophoretic analysis of GLP in coton showed that GLP bands migrated from the level of 95 and 100 kDa to the bands of 50 and 25 kDa after sample treatment with reducing agent (Kim et al., 2004). Similar characteristic of GLP was shown on barley (Zhang et al., 1995) and in canola (Lane, 2002).

Our observations are in line with the previously suggested involvement of cupin fragmentation in plant stress responses, such as response to pathogens (Hamel et al., 1998; Vallelian-Bindschedler et al., 1998), and exposure to salt (Hurkman and Tanaka, 1996) or flooding (Kamal et al., 2015) conditions.

It will be important to further investigate all aspects of cupin-family protein fragmentation in response to plant growth and reproduction in a radio-contaminated environment. Initially it seemed feasible that changes in the abundance of cupin fragments between seed filling in non-radioactive and radio-contaminated environments were detected simply because of the abundance of these proteins in seeds. We now believe that this is not the case because changes in the fragmentation or protein dimers of other abundant proteins (e.g., RuBisCO LSU or the SSP) were not observed in our analyses (Table 1). It is noteworthy that post-fragmentation, the cupin fragments were subsequently stable throughout seed filling (Figure 3; Supplementary Table 1) indicating that fragmentation is not likely to be intermediate in a catabolic or re-allocation pathway.

### Updated working model for flax growth in radio-contaminated environment

**Figure 4:**
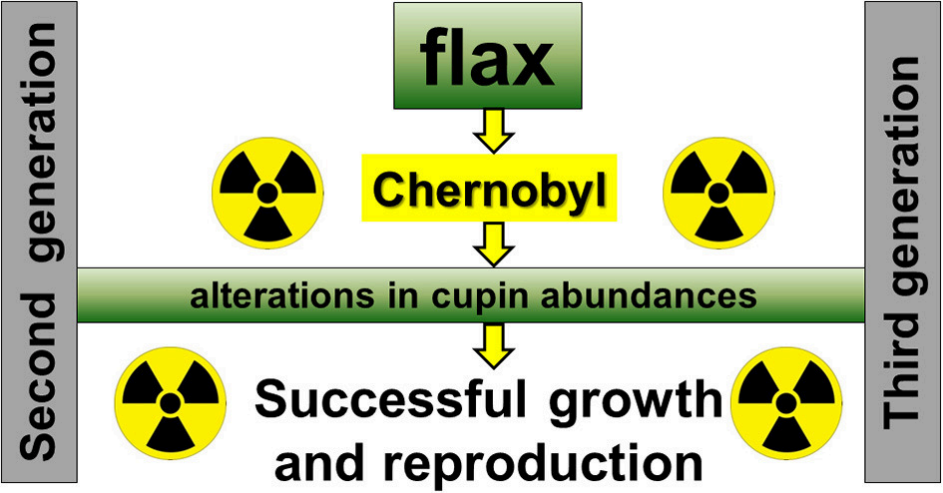
**An Updated model for flax growth in radio-contaminated Chernobyl area**.

Previously, we proposed working model for plant adaptation to a radio-contaminated environment, based on the analysis of two generations of soybean and flax seeds harvested from Chernobyl area (Klubicová et al., 2013). One aspect of this model suggested that alterations in the abundance of proteins associated with carbon assimilation into fatty acid are contributing to the plant success in radio-contaminated environment. However, we did not detected any of these proteins in present study, third flax generation (Table 1). This might be also due to more advanced statistical approach used in this study where ANOVA instead of *T*-test we used. Recently it was suggested that ANOVA is superior over *T*-test for the studies of plant interactions with environment (Brady et al., 2015). However, another aspect of the proposed working model suggested also involvement of SSP in plant success in radio-contaminated environments (Klubicová et al., 2013). Indeed, in the present study we detected a differences in cupin abundance which were previously observed in second-generation flax seeds (Klubicová et al., 2013). Based on this, we propose an updated working model for flax (Figure 4). This model suggests that alterations in cupin abundance during seed filling contribute to the growth and successful reproduction of flax in radio-contaminated Chernobyl area (Figure 4). However, it should be noted that this working model is based on a few differentially abundant proteins identified in this and previous studies and do not provide complex picture on metabolic changes of flax growth in radio-contaminated environment.

## Conclusions

These analyses were performed in order to quantify changes in protein abundance during flax seed development that were conserved across multiple generations of plants grown in the radio-contaminated Chernobyl environment. The logic behind this is to exclude proteome changes which were likely associated with differences between Chernobyl experimental fields or seen only in one generation (Rashydov and Hajduch, 2015). The changes in abundance of cupin fragments were detected jointly in the second and third generations of flax. Based on this an updated working model for growth and reproduction of flax in radio-contaminated environments is suggested. However, to acquire complex understanding of metabolic changes in flax grow in radio-contaminated environment would be necessary to extend our studies for metabolic studies and also to include analyses of plants grown in proximity to the Fukushima Nuclear Power Plant. In future studies we would like to include multiple control and radio-contaminated fields to avoid problems with pseudoreplication (Hurlbert, 1984). If this is not possible, due to restricted access, it might be possible to incorporate an experimental design we have developed specifically for Chernobyl (Rashydov and Hajduch, 2015). This experimental design incorporates one radio-contaminated and two non-radioactive experimental fields, with at least two plant species grown for at least 2 consequent years (Rashydov and Hajduch, 2015).

Data from the present study have been deposited in an easily accessible online database available at www.chernobylproteomics.sav.sk.

## Author contributions

DGa performed 2-DE and KK analyzed 2-DE gels. MD contributed to protein isolation and together with LS conducted MS analyses. DGo performed ANOVA. VB contributed to field work, seed harvest, and protein isolation. JM contributed to writing and editing. NR designed experimental fields, contributed to field work, and seed harvest. MH designed, managed the experiments and performed majority of writing.

### Conflict of interest statement

The authors declare that the research was conducted in the absence of any commercial or financial relationships that could be construed as a potential conflict of interest.

## Supplementary material

Supplementary Table 1 **The statistical analysis of differentially abundant 2-DE spots from developing flax seeds 2 weeks after flowering (WAF) (1A), 4 WAF (1B), 6 WAF (1C), and at seed maturity (1D)**. The table show relative volumes (%V) of biological triplicate analysis, means, standard deviation (SD), and *p*-values. Also displayed is a summary list of the eight 2-DE spots (1E), that were differentially abundant (*p* ≤ 0.001) in at least one developmental stage. The 2-DE spot group ID in non-radioactive and radio-contaminated datasets, developmental stage where the spot was found differentially abundant, and the corresponding *p*-value determined using the ANOVA and Bonferroni correction are shown in 1E.

Supplementary Table 2 **Proteins identified in specific 2-DE spots**.

Supplementary Table 3 **Localization of peptides derived from full-length cupin sequence Lus10003554**.

Supplementary Image 1 **Protein 2-DE gels of developing flax seed proteins harvested 2, 4, and 6 weeks after flowering, and at maturity, from non-radioactive (A) and radio-contaminated (B) experimental fields located in the Chernobyl area**. All gels of biological triplicate analysis used in this study are shown.
