# Supplementary figures and images for: Do Cupins Have a Function Beyond Being Seed Storage Proteins?
Source: Front Plant Sci. 2016 Jan 13;6:1215. doi: 10.3389/fpls.2015.01215 (PMC4711306; doi:10.3389/fpls.2015.01215)

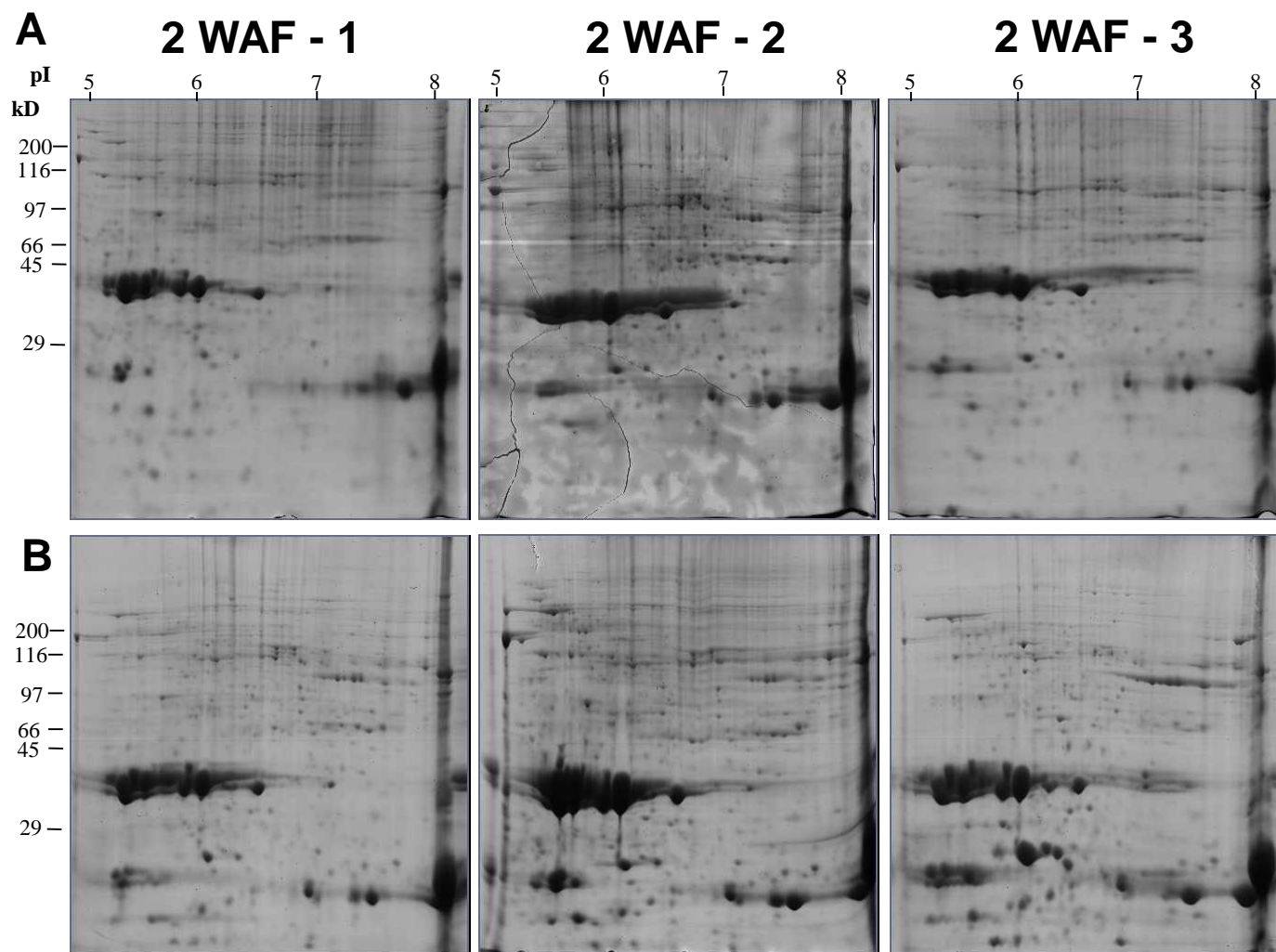

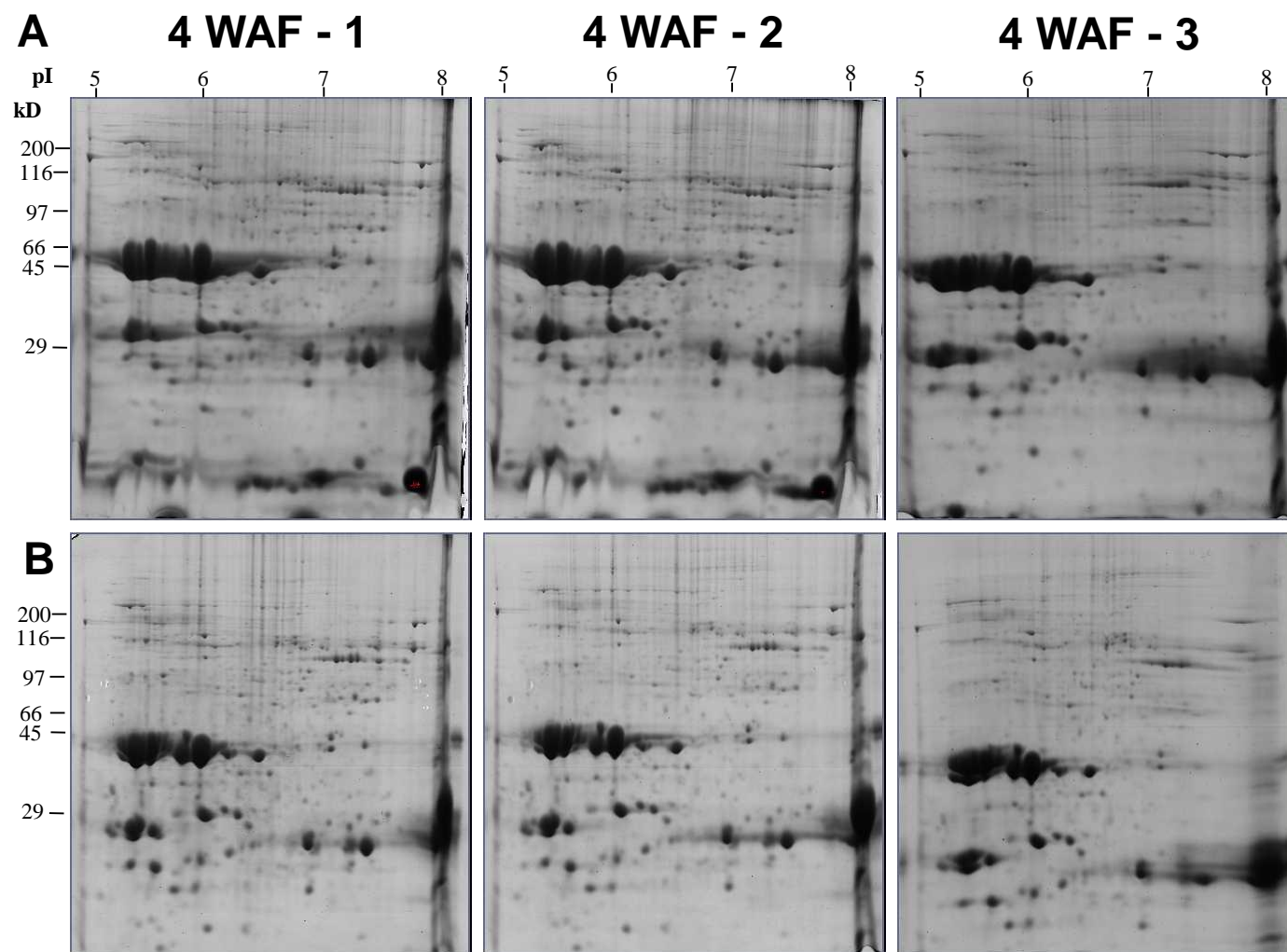

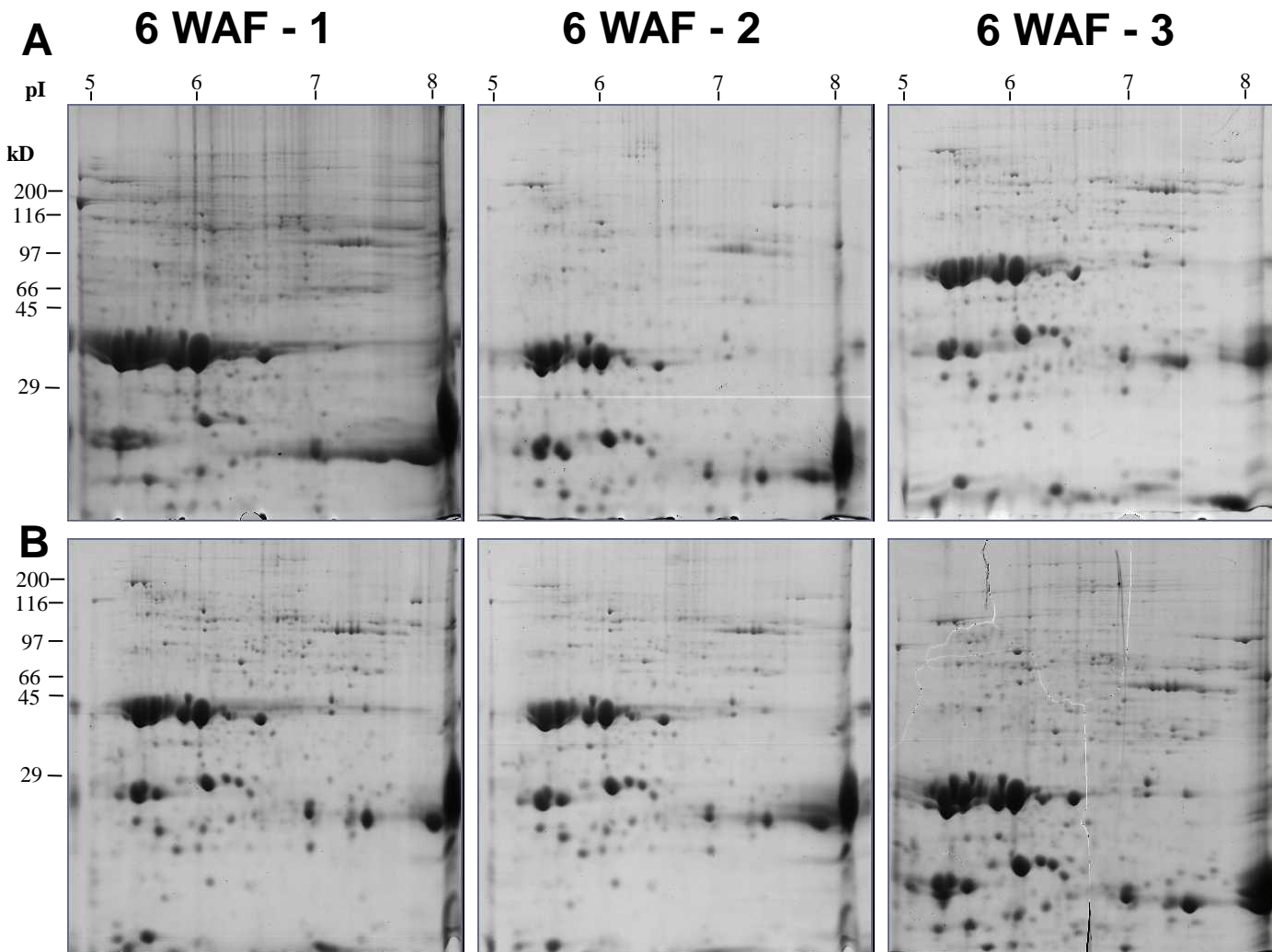

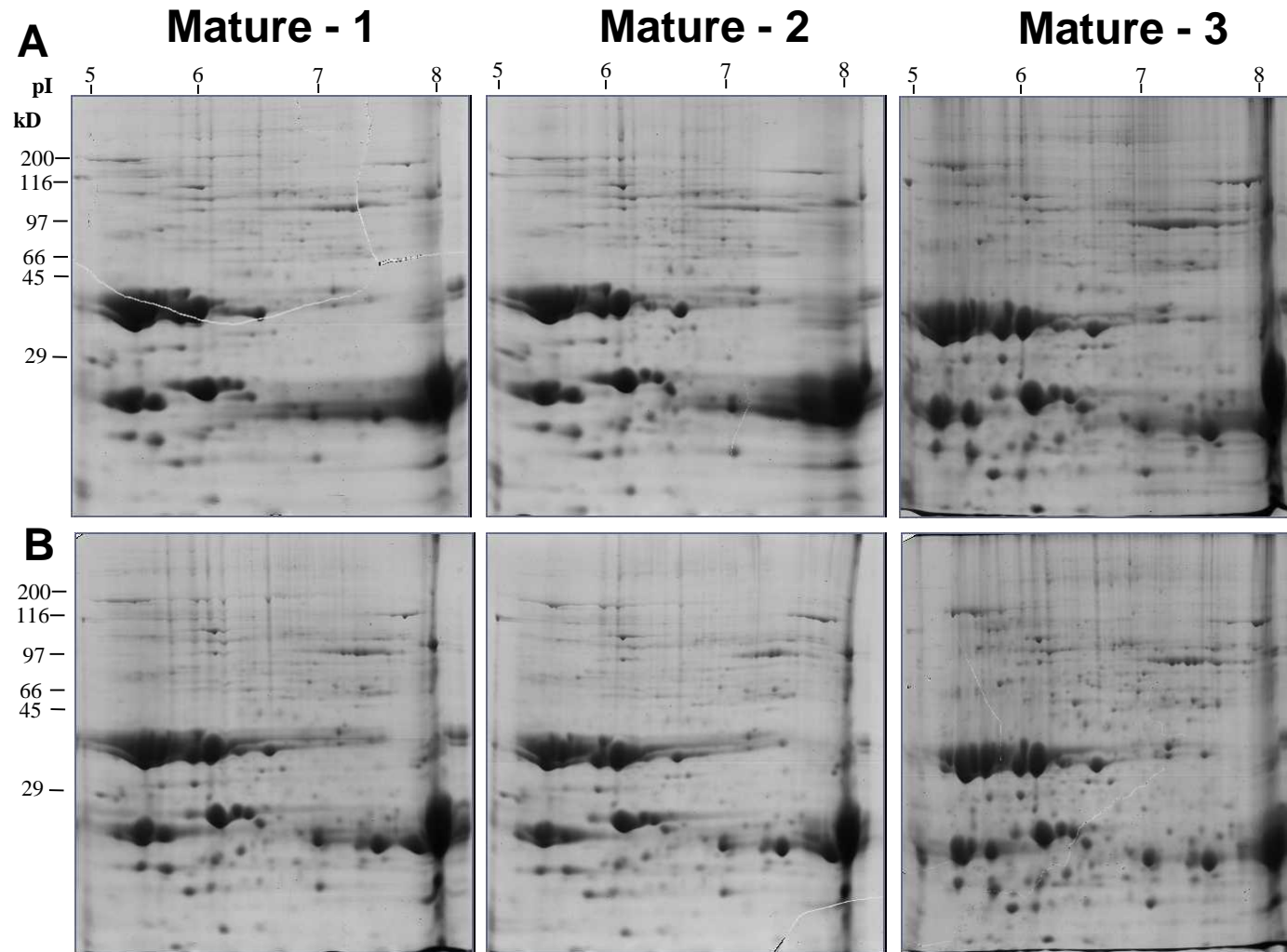

Supplement: Supplementary Image 1 — Protein 2-DE gels of developing flax seed proteins harvested 2, 4, and 6 weeks after flowering, and at maturity, from non-radioactive (A) and radio-contaminated (B) experimental fields located in the Chernobyl area. All gels of biological triplicate analysis used in this study are shown. [file Image1.PDF]
